# Expression of melatonin receptors in trigeminal and sphenopalatine ganglia: potential targets for primary headache disorders

**DOI:** 10.1186/s10194-025-02215-9

**Published:** 2025-12-09

**Authors:** Aida Maddahi, Jacob C. A. Edvinsson, Lars Edvinsson, Diana N. Krause

**Affiliations:** 1 Division of Experimental Vascular Research, Department of Clinical Sciences, Lund University Hospital, Lund, Sweden; 2 Department of Pharmaceutical Sciences, School of Pharmacy & Pharmaceutical Sciences, University of California at Irvine, Irvine, CA USA

**Keywords:** Melatonin, MT1 receptor, MT2 receptor, Trigeminal ganglion, Sphenopalatine ganglion, PCR, Immunohistochemistry, Migraine, Cluster headache, Circadian

## Abstract

**Background:**

Primary headache disorders such as migraine and cluster headache exhibit circadian and circannual variations in attack onset. Melatonin plays a central role in regulating biological rhythms and likely influences the timing of headache attacks. Recent evidence suggests melatonin may be a promising treatment for migraine and cluster headache; however, underlying mechanisms need to be elucidated. Because of the importance of the trigeminovascular system (TVS) and trigeminal-parasympathetic ganglia in these disorders, we proposed that melatonin receptors (MT1 and MT2) are expressed in these pathways.

**Methods:**

The trigeminal ganglion (TG), sphenopalatine ganglion (SPG), dorsal root ganglion (DRG), basilar artery, dura mater and hypothalamus were dissected from adult male and female rats. RT-qPCR and immunohistochemistry were used to assess the expression of mRNA and protein, respectively, for melatonin MT1 and MT2 receptors.

**Results:**

Immunohistochemical analysis showed MT1 and MT2 were differentially expressed in the TG, SPG and DRG. MT1 was widely distributed in the cytoplasm and nuclei of neurons and satellite glial cells (SGCs) in the TG, while MT2 localized mainly in the cytoplasm of neurons and Aδ-fibers. Both MT1 and MT2 co-localized with CGRP and the CGRP receptor component RAMP1. MT2 immunoreactivity was also found in Aδ-fibers throughout the dura mater and was co-expressed with Contactin-associated protein 1(CASPR) at the paranodal regions of Aδ-fibers. No significant sex differences were found in receptor expression in the TG, although mRNA levels of MT1 were approximately twice as high as those for MT2. In SPG, both receptors co-localized with the neuropeptides VIP and PACAP. In cerebral arteries, only MT1 was detected, and it was localized mainly in endothelial and smooth muscle cells.

**Conclusions:**

This study demonstrates that MT1 and MT2 receptors are expressed in the TVS and SPG, key components involved in migraine and cluster headache. Melatonin receptor co-localization with neuropeptides involved in autonomic and sensory neuronal regulation supports a potential mechanism by which melatonin influences headache onset and progression. Together, these findings support a role for melatonin influences in primary headache pathophysiology and point to its potential for novel headache treatment.

**Supplementary information:**

The online version contains supplementary material available at 10.1186/s10194-025-02215-9.

## Introduction

In primary headache disorders, the timing of headache onset exhibits distinct circadian and seasonal rhythms [1–3]. In cluster headache, a rare but highly debilitating pain disorder, there is a clear circadian peak in onset at night between 21:00 and 03:00 as well as circannual peaks of headache clusters that occur in spring and autumn [2, 4]. Migraine attacks also show circadian rhythmicity; a number of studies indicate attack onset is more likely in early morning or late at night [1, 5]. The temporal patterns associated with primary headaches imply chronobiologic influences in the pathophysiology of these disorders [2, 6, 7].

The pineal hormone melatonin (N-acetyl-5-methoxytryptamine) plays a central role in regulating circadian and circannual rhythms and likely influences the timing of headache attacks [8]. Pineal release of melatonin at night is driven by the hypothalamic circadian clock and is regulated by daily and seasonal changes in the light-dark cycle [9]. However, patients with either migraine or cluster headache have low levels of nocturnal melatonin release [2, 10–13], underscoring circadian disruption in these disorders. A number of studies suggest administration of melatonin or related melatonergic analogs may be an effective strategy in the prevention and treatment of migraine and cluster headache [10, 14–19].

The mechanisms by which melatonin influences episodes of migraine and cluster headache are not well understood [20]. Melatonin acts on key brain regions associated with headache initiation, in particular the hypothalamus which contains the primary circadian pacemaker in the suprachiasmatic nucleus [6, 21, 22]. Melatonin also appears to affect spinal and supraspinal pathways involved in pain processing [14]. Of particular interest is potential melatonin action on the peripheral neural systems underlying headache pain and autonomic symptoms, i.e., the trigeminovascular system in migraine and the trigeminal-parasympathetic system in cluster headache. Rhythms in circulating melatonin may promote variations in sensory excitability thresholds that influence symptom onset. Melatonin may also affect signaling by calcitonin gene-related peptide (CGRP), the primary sensory neurotransmitter and target of recent migraine drugs [14, 23].

Melatonin exerts its effects through two G-protein coupled receptors: melatonin receptor 1 (MT1) and melatonin receptor 2 (MT2) [24]. A better understanding of the expression of melatonin receptors in the trigeminovascular and parasympathetic systems may provide insight into how melatonin contributes to the pathophysiology of primary headache disorders and its potential for novel treatment strategies. Therefore, the aims of the current study were to: (1) determine the expression and localization of melatonin receptors in the trigeminal ganglion (TG), sphenopalatine ganglion (SPG), dorsal root ganglion (DRG), dura mater, and cerebral arteries of male and female rats; and (2) examine the co-localization of melatonin receptors with Contactin-associated protein 1 (CASPR), CGRP and its receptors in the TG, as well as with vasoactive intestinal peptide (VIP) and pituitary adenylate cyclase-activating polypeptide (PACAP) in the SPG.

## Experimental procedures

### Animals

All animal procedures in this study followed the guidelines of the European Communities Council (86/609/ECC) and were approved by the Regional Ethical Committee on Animal Research at the University of Lund, Sweden (LU-818–01).The animals were housed under controlled temperature and humidity with free access to water and food ad libitum and were kept under a 12:12-h light: dark cycle (lights on at 10:00 a.m.).

### Tissue preparation

A total of 36 Wistar rats were divided in two groups (24 males weighing approximately 240–300 g; 8–10 weeks and 12 females weighing 200–250 g, 10–12 weeks) and used for immunohistochemistry and RT-qPCR analysis. All animals were anaesthetized using CO_2_ gas and subsequently euthanized by decapitation, between 8:00–9:00 o’clock in the morning. Tissues of interest were carefully dissected: TGs from both male and female rats as well as the SPG (right and left), DRG, basilar artery (BA), dura mater (harvested from the temporal area of the skull) and hypothalamus from male rats. For immunohistochemistry, the dura mater segments were washed in phosphate buffer saline containing 0.3% Triton X-100 (PBST) and spread out on microscope slides (Super frost, ThermoFisher) to dry. The SPGs, TGs, DRGs, dura mater and BA were fixed in 4% paraformaldehyde (PF) in phosphate buffer saline (PBS, Sigma Aldrich, pH 7.2) for 4 hours at room temperature. All fixed tissues were cryo-protected with a Sörensen’s phosphate buffer (pH 7.2) gradient containing 10% and 25% sucrose overnight. The TGs, SPGs, DRG and BAs were embedded in an egg albumin-based protein medium or in Tissue- Tek (O.C.T. Compound, Sakura, Poland) and sectioned at a thickness of 10 µm using a cryostat (Microm Cryo Star HM 560). Finally, the sections were collected on microscope slides (Superfrost^TM,^ Merck Chemicals and Life Science, Sweden) and stored at − 20 °C until use. For RT-qPCR, the TGs and SPGs (left and right from each rat), and hypothalamus were dissected and immediately fresh frozen in liquid nitrogen. Then they were collected in a 2 ml Eppendorf tube and stored at − 80 °C for further analysis.

### RNA isolation and RT-qPCR

A total of 18 rats (12 males and 6 females) were used for RT-qPCR. The hypothalamus and SPG were dissected from 6 male rats, while TGs were obtained from another 6 male and 6 female rats. All tissues were carefully dissected and immediately frozen in liquid nitrogen for RNA extraction. All RNA extraction was performed using RNeasy® Plus Mini kit (Qiagen, Hilden, Germany) in accordance with the manufacturer’s protocol. Total RNA concentration was determined using a GeneQuant Pro spectrophotometer (Amersham Pharmacia Biotech, Uppsala, Sweden). A ratio of sample absorbance at 260/280 nm in the range of 1.8 to 2 was considered acceptable. First-strand cDNA was prepared from 1 µg of total RNA (from TG, SPG and hypothalamus) in a 20 µL reverse transcript reaction using Superscript® III First-Strand Synthesis Super Mix (Invitrogen, Carlsbad, CA, USA). A reverse transcription negative control to detect the genomic DNA was performed simultaneously for each sample and underwent the same procedures but without Superscript III Reverse Transcriptase (RT enzyme). The cDNA obtained was diluted to a total volume of 80 µL and stored at −20 °C. The primer sequences were specific for the genes of interest and were designed for Rattus norvegicus, using Primer Express 3.0 software (PE Applied Biosystems, Foster city, CA, USA), and synthesized by TAG Copenhagen A/S (Copenhagen, Denmark). The housekeeping gene glyceraldehyde-3-phosphate dehydrogenase (GAPDH) was used as a reference gene, and the gene expressions were normalized versus that gene. Primers had the following sequences:

MT1 (forward; 5`-CAGTACGACCCCCGGATCTA −3`, reverse; 5` -GGCAATCGTGTACGCCG −3`); MT2 (forward; 5`- ATGTTCGCAGTGTTTGTGGTTT −3`, reverse; 5`-ACTGCAAGGCCAATACAGTTGA- 3`); GAPDH (forward; 5`-CTGCACCACCAACTGCTTAGG −3`, reverse; 5´-TCAGCTCTGGGATGACCTTGC- 3`).

The RT-qPCR was performed in 20 µL reaction consisting of 2 µL diluted cDNA, 0.5 µM of each primer, 10 µL Fast SYBR™ Green Master Mix (Applied Biosystems, CA, USA), and 7 µL RNase free water in a Step One Plus Real Time PCR System (Applied Biosystems, CA, USA) with the following thermal profile: Holding stage at +50 °C for 2 min, +95 °C for 10 min, followed by 40 PCR cycles at +95 °C for 15 s and +60 °C for 1 min. Each sample was examined in duplicate, and a blank control (without template) was used in all experiments. After amplification, a melting curve analysis was performed to confirm specificity of primers annealing and to verify that each primer pair generated only one PCR product of expected size.

### Immunohistochemistry

Sections of TG from male and female rats, along with dura mater, BA, DRG and SPG from male rats, were washed and permeabilized in PBS containing 0.3% Triton X-100 (PBST) for 15 minutes. Thereafter, the tissues were blocked for non-specific binding of antibodies for 1 hour at room temperature in blocking solution containing PBST, 1% bovine serum albumin (BSA), and 5% normal serum. The sections were incubated overnight at 4 °C in moisturized chambers with primary antibodies (for antibodies detail see Table 1). The next day, the sections were washed in PBST for 3 × 15 minutes and incubated with secondary antibodies (for details, see Table 1) for 1 hour at room temperature and kept in the dark to minimize loss of fluorescence. All antibodies were diluted in PBST containing 1% BSA. The sections were subsequently washed with PBST for 3 × 15 minutes and mounted with anti-fading Vectashield mounting medium containing 4’, 6-diamidino-2-phenylindole (DAPI) (Vector Laboratories, Burlingame CA, USA). The procedure described was performed in triplicate for each animal to ensure reproducibility. Further, negative controls were included by omitting the primary antibody to evaluate non-specific secondary antibody binding. Immunoreactivity was visualized using an epifluorescence microscope (Nikon 80i; Tokyo, Japan) at the appropriate wavelengths and photographed with an attached Nikon DS-2 Mv camera. Images were processed using Adobe Photoshop CS3 (v0.0 Adobe Systems, Mountain View, CA).

**Table 1:** Details of primary and secondary antibodies are used for immunohistochemistry

Primary antibodies				
Name and product code	Dilution	Host	Supplier	RRID
CGRP (ab81887)	1:100	Mouse	Abcam, Cambridge, UK	AB_1658411
RAMP1(844)	1:100	Goat	Merck & Co., Inc.,	—–
MT1 (PA5-75749)	1:100	Rabbit	USA ThermoFisher Scientific, MA, USA	AB_2719477
MT1(sc-390328)	1:50	Mouse	Santa Cruz Biotechnology, USA	AB_2810849
MT1 (AMR-031)	1:200	Rabbit	Alomone Labs, Jerusalem, Israel	AB_11218959
MT1 (bs-0027 R)	1:200	Rabbit	Bioss Inc, Woburn, USA	AB_10856893
MT2 (ab203346)	1:200	Rabbit	Abcam, Cambridge, UK	AB_2783824
MT2 (AMR-032)	1:200	Rabbit	Alomone Labs, Jerusalem, Israel	AB_2340995
CASPR (MABN69)	1:100	Mouse	Millipore, Temecula, USA	AB_10806491
**Secondary antibodies**				
Name	Dilution	Against	Supplier	RRID
Alexa 594	1:200	Goat	Thermo Scientific, IL, USA	AB_2762828
Alexa 594	1:200	Mouse	Thermo Scientific, IL, USA	AB_2535789
Alexa 488	1:100	Rabbit	Thermo Scientific, IL, USA	AB_2535792
Alexa 594	1:200	Rabbit	Thermo Scientific, IL, USA	AB_141637

To further validate our results, four different antibodies were selected to detect expression of MT1, and two different antibodies were chosen to detect expression of MT2 (Table 1). In a recent review of melatonin receptors, the MT1 and MT2 antibodies from Alomone Labs, in particular, were considered to be reliable and specific [25].

### Double immunohistochemistry

Double immunohistochemistry was performed using antibodies against MT1 or MT2 in combination with antibodies to CGRP, RAMP1 (receptor activity-modifying protein-1), VIP, PACAP or CASPR (marking juxta- and paranodal regions of myelinated axons). All procedures were the same as described above. Since the CLR, MT1, and MT2 antibodies belong to the same host species, double staining was not performed.

### Cell counting

Cell counting was performed to semi-quantify the expression of MT1 and MT2 in TG, following the method described by Warfvinge et al. [26]. Three slides with three sections on each were used for measurements. Counting of cells which had visible nuclei was performed in the central part of pooled ophthalmic-maxillary and mandibular areas. Due to the risk of artefactual fluorescence, counting of neurons close to the TG surface was not performed. Images were taken of the screen (0.75 mm^2^) at 10 ×magnification. All cells in this area, including negative and immunoreactive cells, were counted. The mean percentage of positive neurons in 3 slides/rat, from all rats (*n* = 6) was used for analysis.

### Calculation and statistical analyses

RT-qPCR data were analyzed with the comparative cycle threshold (Ct) method [27]. The Ct value of GAPDH mRNA was used as the reference gene to normalize the expression levels of the target genes MT1 and MT2 in each sample. The relative expression of each target gene was calculated using the formula: X0/R0 = 2^(CtR-CtX)^ = 2^-ΔCt^, where X_0_ is the amount of target mRNA, R_0_ is the amount of reference gene mRNA, Ct_R_ is the Ct value of the reference gene, Ct_X_ is the Ct value of the target gene, and ΔCt = Ct_X_-Ct_R_.

All statistical analyses were performed using Graph Pad Prism 9. Statistical significance for RT-qPCR and immunohistochemistry was determined using Mann-Whitney or Student’s t-test. Data were expressed as mean ± standard error of the mean (SEM), *n* refers to the number of rats, *p < 0.05, **p < 0.01 and ***p < 0.001 were considered significant.

## Results

### Gene expressions of MT1 and MT2 in rat TG, SPG and hypothalamus

Expression of the genes for MT1 (Mtnr1a) and MT2 (Mtnr1b) in male and female rat TG was determined by real-time PCR analysis of tissue mRNA. In each qPCR experiment, either a no template control (NTC, water control) or a minus RT control was included, and there were no signs of contamination, primer-dimer and/or genomic DNA in the samples. The expression of the house keeping gene GAPDH was used to normalize gene expression.

**Fig. 1:**
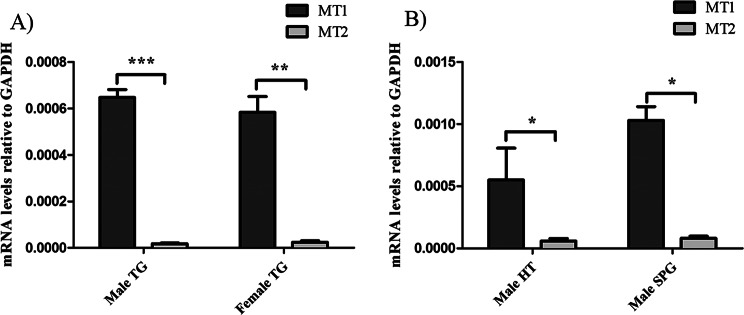
Expression of MT1 and MT2 genes in rat TG, SPG and hypothalamus. (**A**) Comparison of MT1 and MT2 mRNA levels in the TG of male and female rats shows that MT1 expression is significantly higher than MT2. (**B**) In both the hypothalamus (HT) and SPG of male rats, MT1 mRNA levels are also significantly higher than MT2. Data are presented as mean ± SEM, statistical significance indicate as ***p < 0.001, **p < 0.01, *p < 0.05, and *n = 6*

The results in Fig. 1A show that both MT1 and MT2 genes are expressed in the TG, however the level of mRNA for MT1 was much higher than that for MT2. The pattern of receptor expression appears similar in the TG of males and females. Male TG had the highest MT1 expression at approximately 0.0007 relative to GAPDH, whereas MT1 expression in the female TG was around 0.0006 relative to GAPDH.

Similarly, mRNA expression for MT1 and MT2 receptors was found in the male SPG, with MT1 expression much higher than that for MT2 (Fig. 1B). The hypothalamus from male rats was also analyzed as a positive control. Expression of mRNA for both MT1 and MT2 receptors was detected, with significantly higher levels of MT1 mRNA relative to MT2 mRNA (Fig. 1B).

### Immunohistochemistry

#### Expression and localization of MT1 and MT2 in TG

**Fig. 2:**
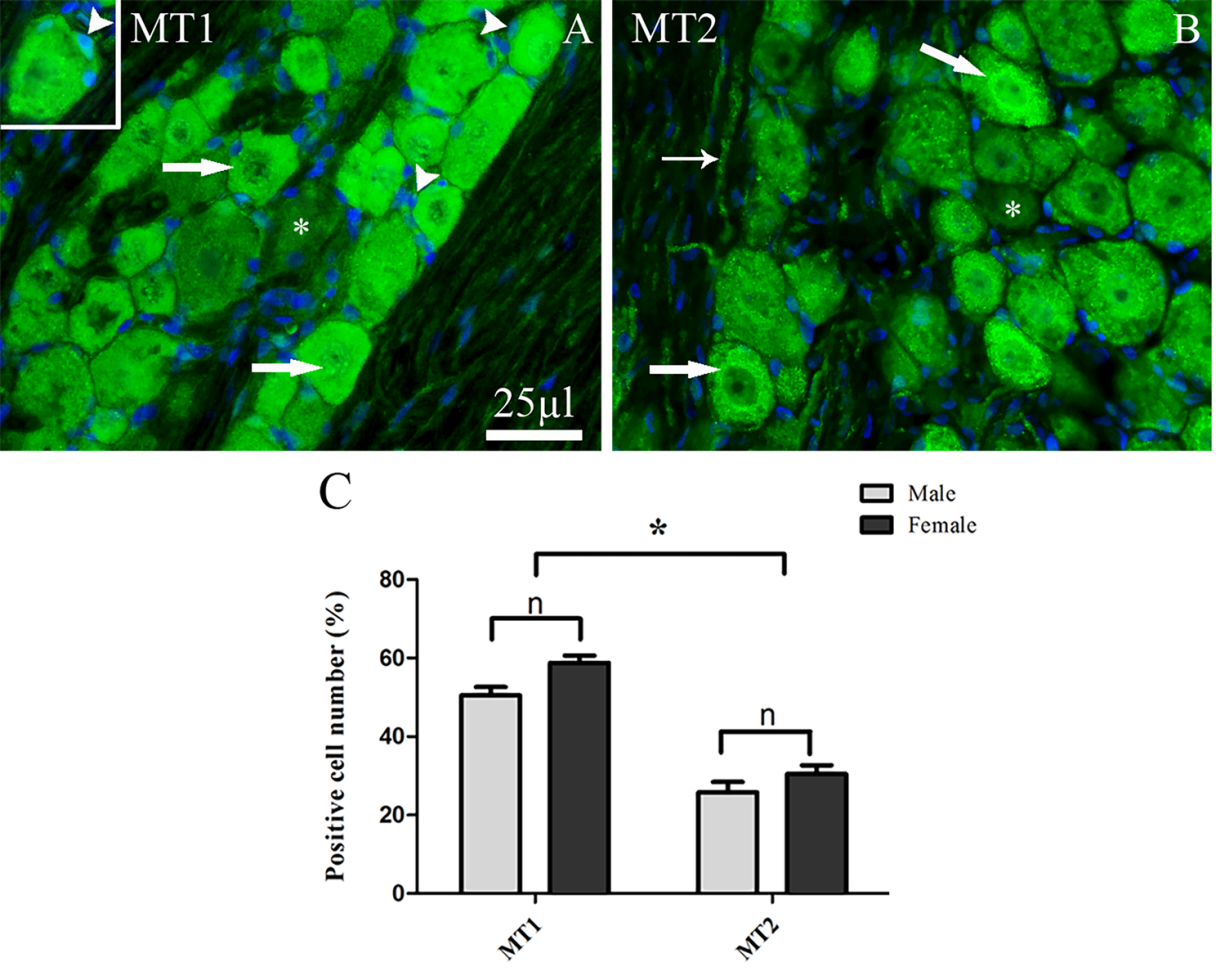
MT1 and MT2 immunoreactivity in TG. (**A**) positive MT1 immunoreactivity was observed in the cytoplasm and nuclei of TG neurons (thick arrows) and in SGCs (arrowheads). (**B**) MT2 immunoreactivity was observed predominantly in the cytoplasm of neurons (thick arrows), in some cases in their nuclei (thick arrows), and in Aδ-fibers (thin arrows). Negative cells are indicated by asterisks in both (**A**) and (**B**). Blue color represents nuclei staining with DAPI. (**C**) Bar graphs show the number of MT1 and MT2 immunoreactive TG neurons in male and female rats. There was no significant difference in MT1 or MT2 protein expression between sexes. However, the number of MT1-immunoreactive cells was approximately twofold higher than that of MT2 in both males and females. Data are presented as the mean ± SEM, *n* = 6 and *p < 0.05

To confirm the reliability of our results, four different antibodies against MT1 from separate suppliers were tested. The sc-390328 antibody from Santa Cruz showed non-specific and high intensity staining limited to only cell membranes and fiber walls, with no labeling of neuronal cell bodies or fibers. In contrast, antibodies from Alomone lab, Thermo Scientific, and Bioss produced comparable results, showing cytoplasmic and nuclear staining in neurons with only very weak or absent fiber staining. Among these, the Bioss antibody provided the clearest and most consistent staining and was therefore used for illustration in figures. As shown in Fig. 2A, MT1 protein was detected in both the cytoplasm and nuclei of neurons of various sizes and in the satellite glial cells (SGCs). We calculated the number of MT1-immunopositive neurons and observed that 55–60% of all TG neurons expressed MT1 immunoreactivity (Fig. 2C).

Two different antibodies from Abcam and Alomone labs were used to detect MT2, and both produced similar results. The Abcam MT2 antibody was selected to visualize the results in the figures. As shown in Fig. 2B, MT2 immunoreactivity was detected mostly in the cytoplasm of medium to large -sized neurons and in the Aδ-fibers. In addition, MT2 immunoreactivity was found to some extent in the nucleus. The results showed that 25–30% of all TG neurons expressed MT2 immunoreactivity (Fig. 2C).

MT1 and MT2 protein expression appeared similar in the TG of male and female rats. In both sexes, the number of MT1 immunoreactive cells was twofold higher than that for MT2 receptors (Fig. 2C).

### Co-localization of MT1 with CGRP and RAMP1 in the TG

**Fig. 3:**
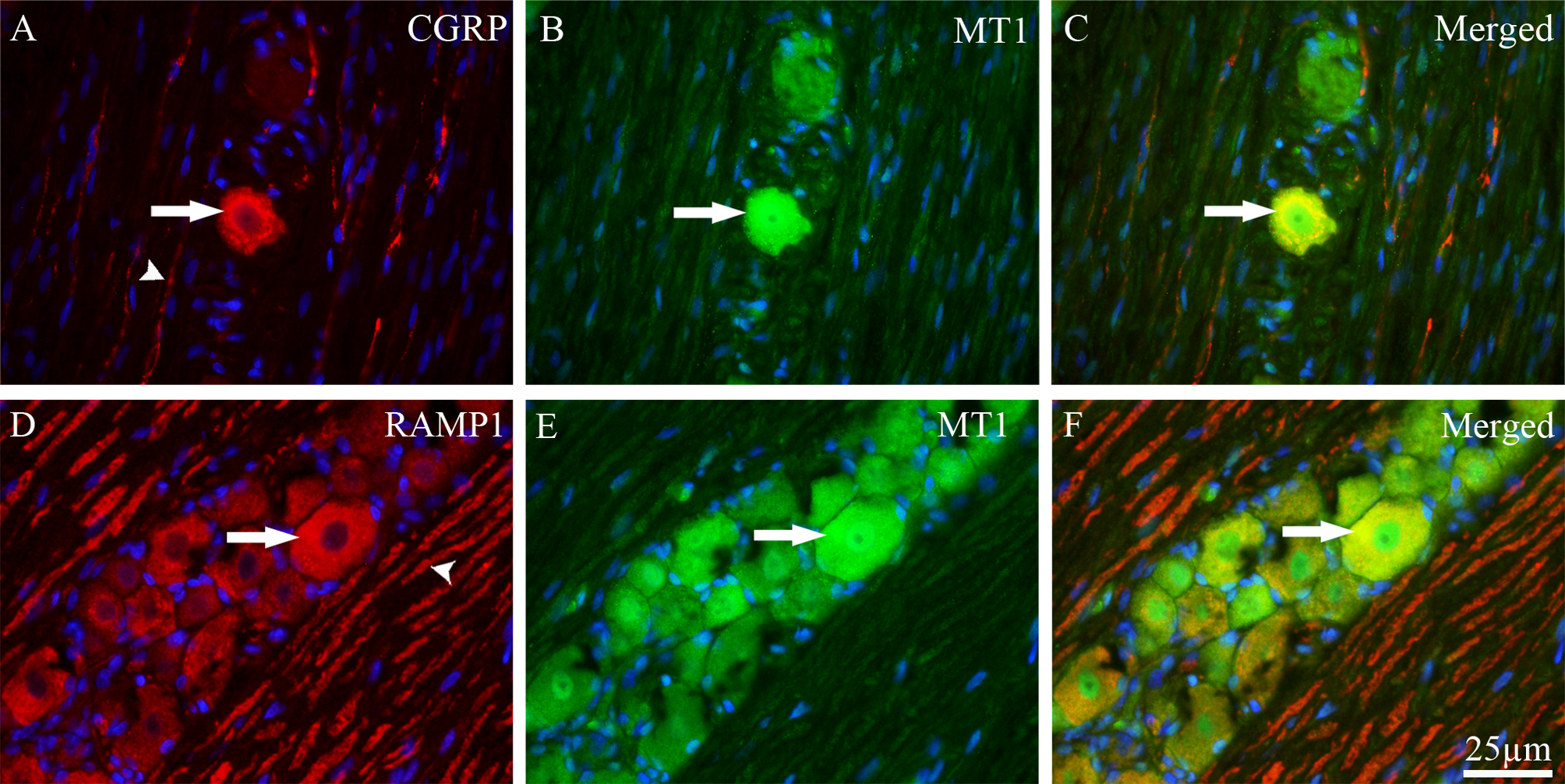
Double immunohistochemistry of MT1 with CGRP or RAMP1 in TG. (**A**) CGRP (red fluorescence) was expressed in the cytoplasm of TG neurons (thick arrow) and in C- fibers (arrowhead). (**B**) MT1 (green fluorescence) was expressed in both cytoplasm and nuclei of TG neurons (thick arrow). (**C**) The merged image shows that MT1 was co-localized with CGRP in the cytoplasm of small to medium-sized neurons (yellow, thick arrow). (**D**) RAMP1 (red fluorescence) was expressed in the cytoplasm of medium to large- sized TG neurons (thick arrow) and in Aδ-fibers (arrowhead). (**E**) MT1 (green fluorescence) was expressed in both cytoplasm and nuclei of TG neurons (thick arrow). (**F**) Their co-localization (yellow) is seen in the merged image. MT1 was co-localized with RAMP1 in the cytoplasm of medium to large -sized neurons (thick arrow). Blue represents nuclei staining with DAPI

MT1 receptors and CGRP were co-localized in the cytoplasm of approximately 10% of small to medium-sized neurons (Fig. 3A–C). No co-localization was observed between MT1 and CGRP in nerve fibers or glial cells.

MT1 is also co-localized with RAMP1, an integral component of the CGRP receptor, in about one-third of the medium-sized neurons. However, no co-localization of MT1 was found with RAMP1 in the Aδ-fibers (Fig. 3D–F).

### Co-localization of MT2 with CGRP, RAMP1 or CASPR in the TG

**Fig. 4:**
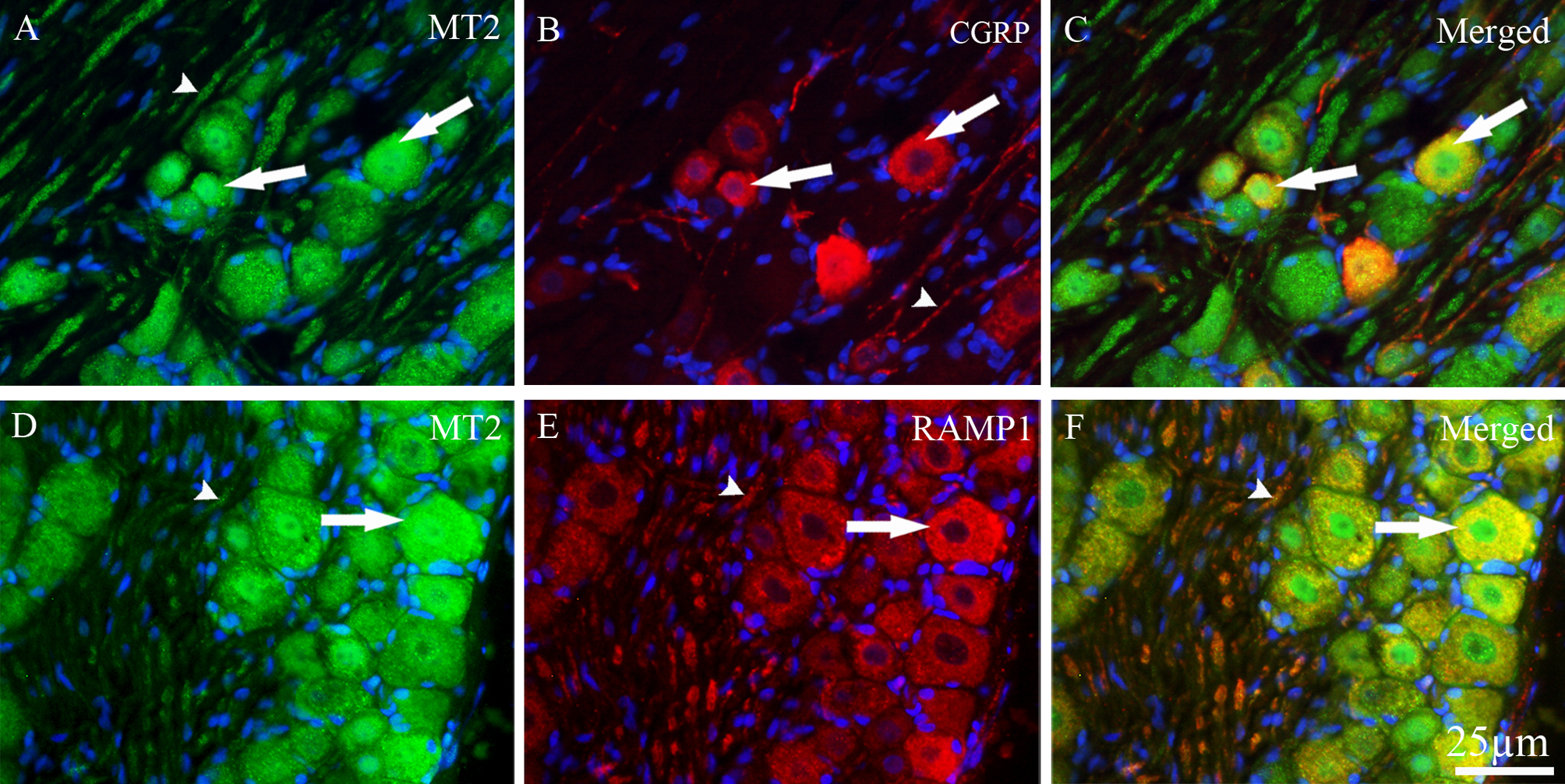
Double immunohistochemistry of MT2 with CGRP or RAMP1 in TG. (**A** and **D**) MT2 immunoreactivity (green fluorescence) was observed in cytoplasm of TG neurons (thick arrows), and in Aδ- fibers (arrowheads). (**B**) CGRP (red fluorescence) was expressed in small to medium-sized TG neurons (thick arrows) as well as in C-fibers (arrowhead). (**C**) MT2 co-localized with CGRP (yellow-orange) in the cytoplasm of small to medium-sized neurons (thick arrows). (**E**) RAMP1 (red fluorescence) was expressed in the cytoplasm of medium to large- sized TG neurons (thick arrow) and in Aδ-fibers (arrowhead). (**F**) The merged imaged showed that MT2 was co-localized (yellow) with RAMP1 in the cytoplasm of medium to large-sized neurons (thick arrow) as well as in the Aδ- fibers (arrowhead)

We examined the co-localization of MT2 with CGRP and RAMP1 in TG and found that MT2 receptors and CGRP were co-localized in approximately 15% of small to medium-sized neurons (Fig. 4A–C). CGRP immunoreactivity was also observed in thin fibers (C-fibers) (Fig. 4B), whereas RAMP1 immunoreactivity was detected in thick nerve fibers (Aδ-fibers) (Fig. 4A). No co-localization between MT2 and CGRP was observed in nerve fibers. MT2 and RAMP1 were co-localized in approximately 25% of the medium to large-sized TG neurons, as well as in Aδ-fibers (Fig. 4D–F).

**Fig. 5:**
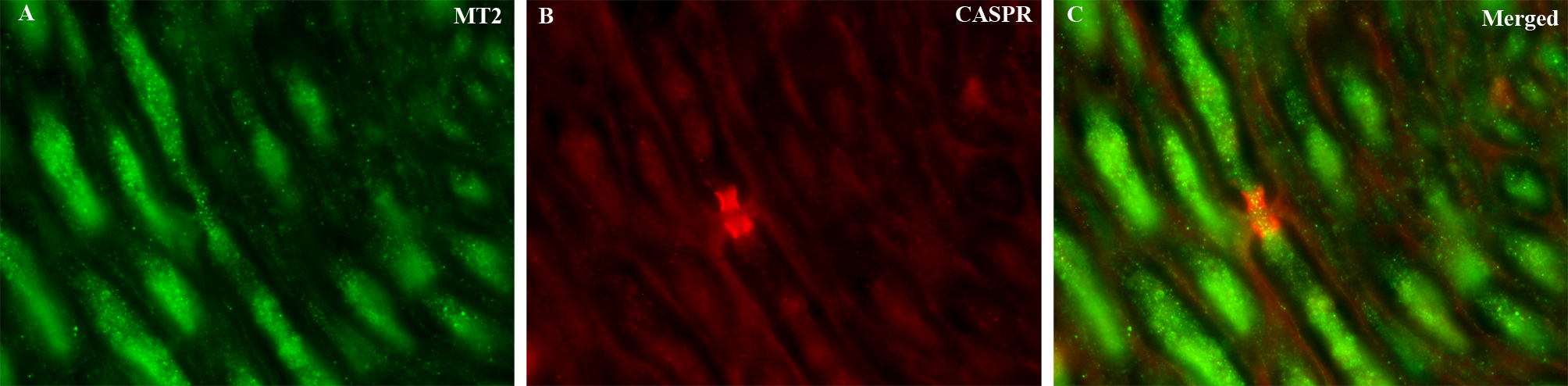
Double immunohistochemistry of MT2 and CASPR in trigeminal Aδ-fibers. (**A**) MT2 receptors (green) were found in the Aδ-fibers. (**B**) CASPR immunoreactivity (red) was used to label the paranodal regions of nodes of Ranvier. (**C**) MT2 and CASPR were co-expressed in Aδ-fibers. The blue color represents nuclei staining with DAPI

To further examine MT2 distribution within the Aδ axons, double staining was performed with CASPR, a membrane protein expressed in the juxta- and paranodal regions flanking the nodes of Ranvier in myelinated fibers (Fig. 5B). MT2 receptor protein was co-expressed with CASPR at the nodes of Ranvier (Fig. 5C). This expression was specifically along the Aδ-fibers and indicates that MT2 is expressed in the nodal regions of myelinated trigeminal axons.

### CGRP and MT2 expression in the dura mater

The trigeminal nerve, including C- and Aδ-fibers, innervates the dura mater, which mainly consists of connective tissue and blood vessels originating from the external carotid artery that distributes blood intracranially via the middle meningeal artery.

**Fig. 6:**
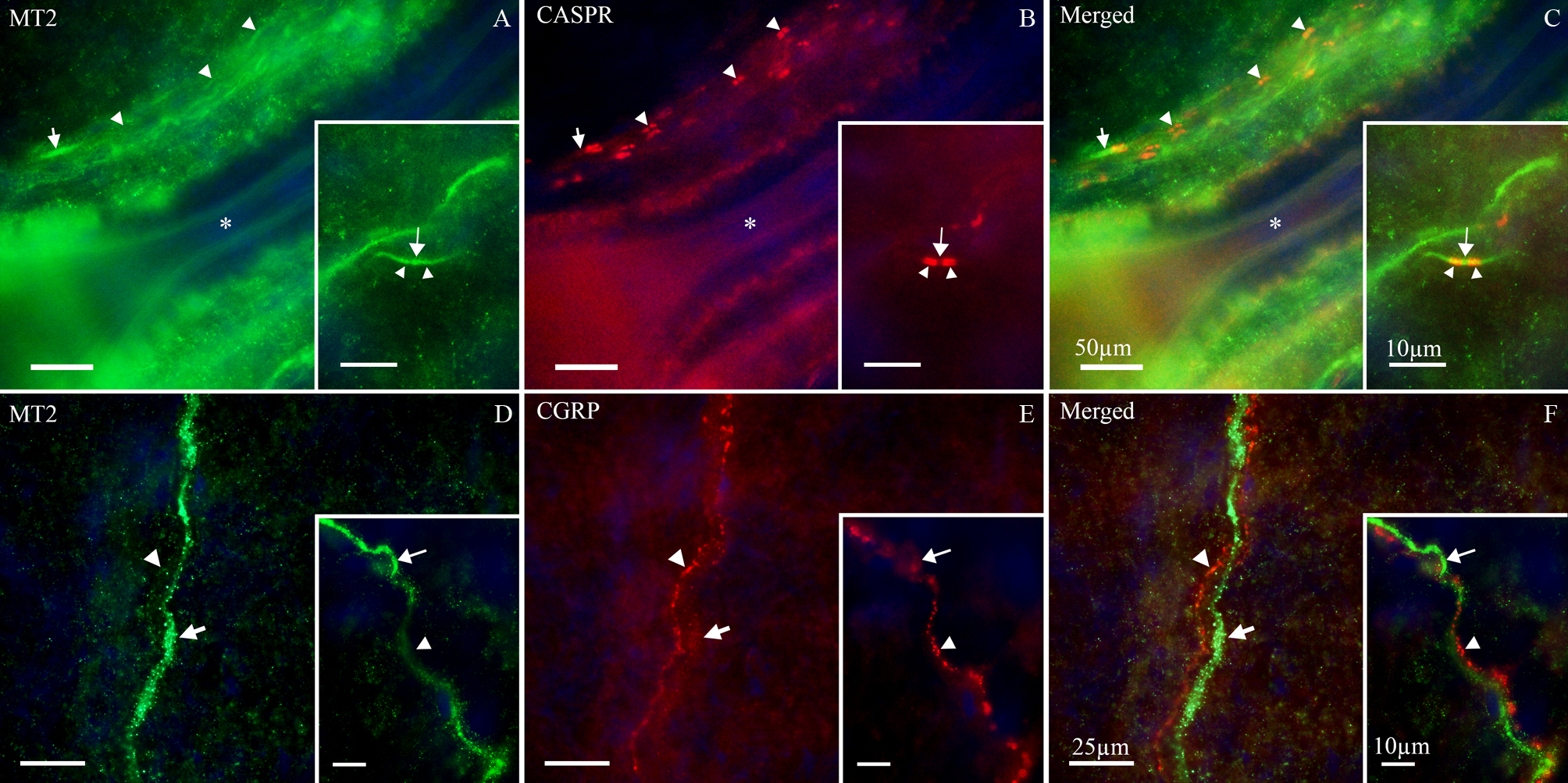
Double immunohistochemistry of MT2 with CASPR and CGRP in rat dura mater. (**A** and **D**) Thin MT2-immunoreactive axons (green, arrows) were observed in the rat dura mater. (**B**) These fibers were identified as Aδ-fibers based on CASPR immunoreactivity (red) showing the characteristic bowtie-shaped paranodal pattern (arrowheads). (**A**-**C**) Fiber bundles were often seen flanking dural blood vessels (asterisks). (**C**) MT2 and CASPR were observed to be co-expressed in the same Aδ-fibers. (**E**) CGRP immunoreactivity (red, arrowheads) was detected in c-fibers throughout the rat dura mater in a granular, pearl-like pattern. (**F**) Merged images show intertwined C- and Aδ-fibers, expressing CGRP and MT2 respectively, projecting throughout the dura mater

Based on the finding of MT2 receptors in nerve fibers within the TG, we also examined MT2 expression in the rat dura mater. Because the dura is relatively dense, it can be more difficult to penetrate with antibodies and often exhibits higher background staining; therefore, MT2 immunoreactivity was confirmed by co-labeling with the CASPR (Fig. 6A–C). Thin MT2-immunoreactive axons were observed throughout the dura mater, many of which co-expressed the characteristic bowtie-shaped CASPR pattern indicative of Aδ-fibers. CGRP immunoreactivity was detected in thin C-fibers distributed throughout the dura mater (Fig. 6E). MT2 and CGRP did not co-localize within the same fibers. However, CGRP positive C-fibers and MT2 positive Aδ-fibers were often found in close proximity, forming intertwined bundles projecting through the dura mater (Fig. 6F). No expression of MT1 was observed in sensory fibers of the dura mater.

### Expression and localization of MT1 and MT2 in the SPG and their co-localization with VIP and PACAP

**Fig. 7:**
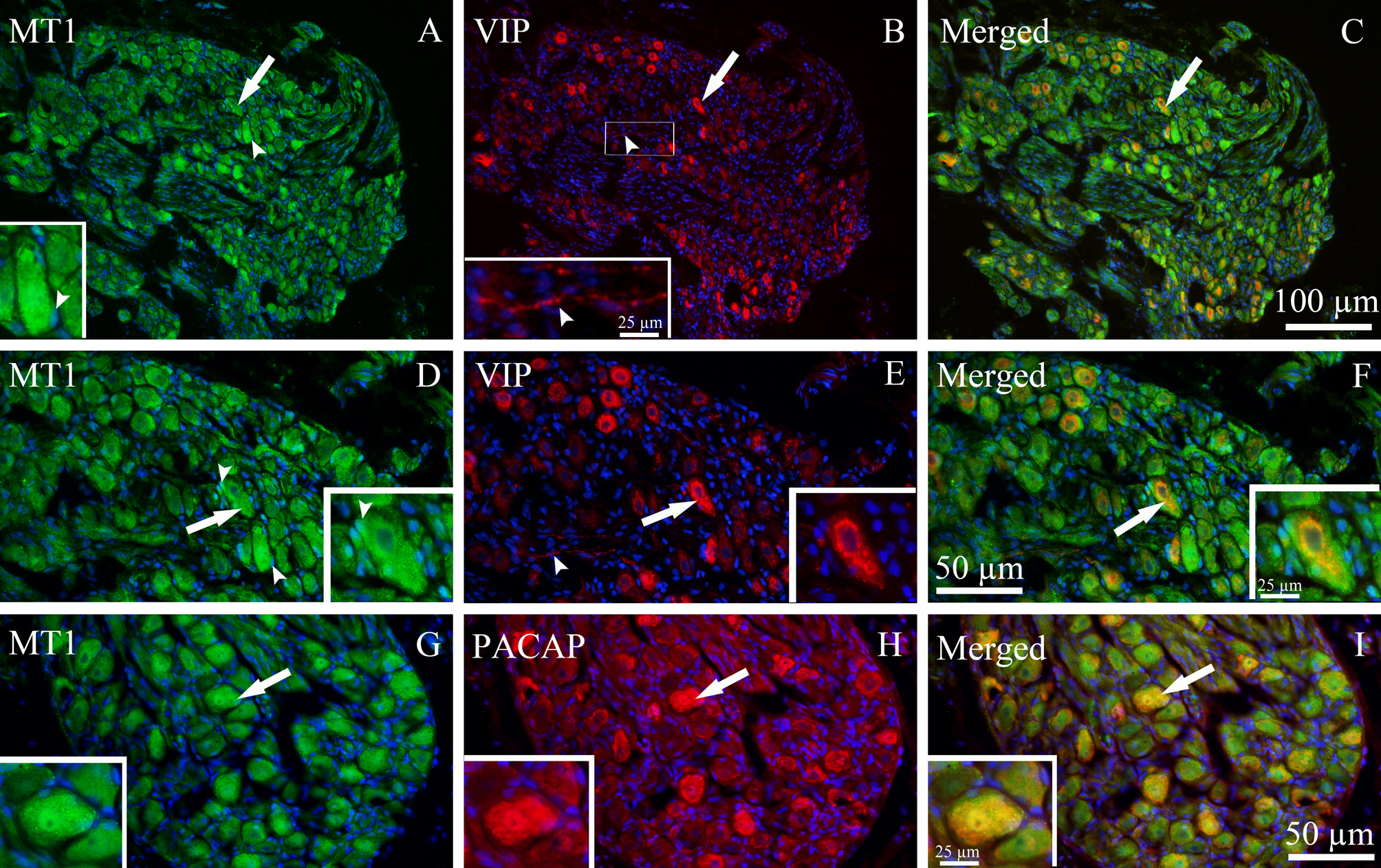
Localization of MT1 immunoreactivity in the SPG and its co-localization with VIP and PACAP. (**A**, **D** and **G**) MT1 immunoreactivity (green) was observed in both the cytoplasm and nuclei of SPG neurons (thick arrows), as well as in SGCs (arrowheads). (**B** and **E**) VIP immunoreactivity (red) was detected in the cytoplasm of SPG neurons (thick arrows) and in a few C-fibers (arrowhead). (**C** and **F**) MT1 was co-localized with VIP in the cytoplasm of some SPG neurons (yellow-orange, thick arrows). **D**, **E** and **F** are higher-magnification views of images **A**, **B** and **C**, respectively. (**H**) PACAP immunoreactivity (red) was found in both the cytoplasm and nuclei of some SPG neurons (thick arrow). (**I**) MT1 was co-localized with PACAP in SPG neurons as shown in merged images (yellow/orange, thick arrow)

**Fig. 8:**
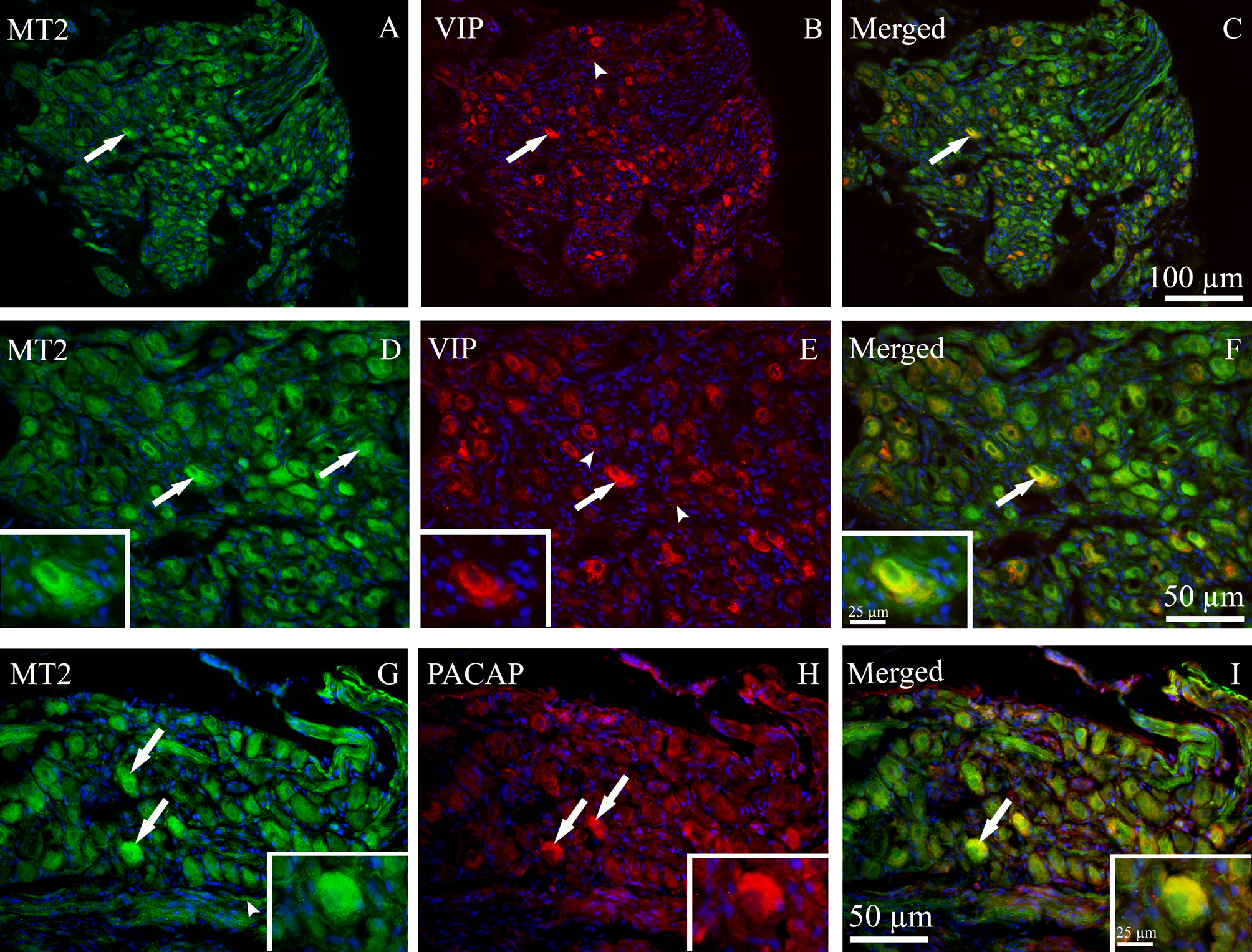
Localization of MT2 immunoreactivity in the SPG and its co-localization with VIP and PACAP. (**A**, **D** and **G**) MT2 immunoreactivity (green) was observed in the cytoplasm of neurons (thick arrows), and in fibers (arrowhead). (**B** and **E**) VIP immunoreactivity (red) was detected in the cytoplasm of many medium-sized neurons (thick arrows) and in a few fibers (arrowheads). (**C** and **F**) MT2 was co-localized with VIP in the cytoplasm of medium sized neurons (yellow-orange, thick arrows). **D**, **E** and **F** are higher-magnification views of images **A**, **B** and **C**, respectively. (**H**) PACAP immunoreactivity (red) was found in the cytoplasm of SPG neurons (thick arrows). (**I**) Double staining of MT2 and PACAP showed that MT2 was co-localized with PACAP in the cytoplasm of SPG neurons (yellow-orange, thick arrow)

MT1 and MT2 receptors were both expressed in SPG (Figs. 7 and 8). MT1 immunoreactivity was observed in the cytoplasm and nucleus of SPG neurons of all sizes, as well as in the SGCs (Figs. 7A, 7D and 7G).

MT2 immunoreactivity was predominantly located in the cytoplasm of medium to large-sized neurons and nerve fibers, as illustrated in Figs. 8A, 8D and 8G.

Localization of the neuropeptides VIP and PACAP was also determined in the SPG. VIP immunoreactivity was primarily found in the cytoplasm of many medium-sized neurons and in a few C-fibers (Figs. 7B, 7E, 8B and 8E). In both neurons and fibers, the immunoreactivity displayed a granular-like pattern. PACAP immunoreactivity also was mainly detected in the cytoplasm of medium to large-sized neurons, and in some cases, also in the nuclei of large-sized neurons (Figs. 7H and 8H).

Both MT1 and MT2 receptors were expressed in VIP and PACAP containing SPG neurons. MT1 and VIP immunoreactivity was co-localized in the cytoplasm of SPG neurons (Figs. 7C and 7F). MT1 and PACAP also showed co-localization in both the cytoplasm and the nuclei of numerous neurons (Fig. 7I). MT2 was co-localized with both VIP and PACAP in the cytoplasm of SPG neurons. However, there was no co-localization of MT2 and VIP in nerve fibers (Figs. 8F and 8E).

### Expression and localization of MT1 and MT2 in the DRG

**Fig. 9:**
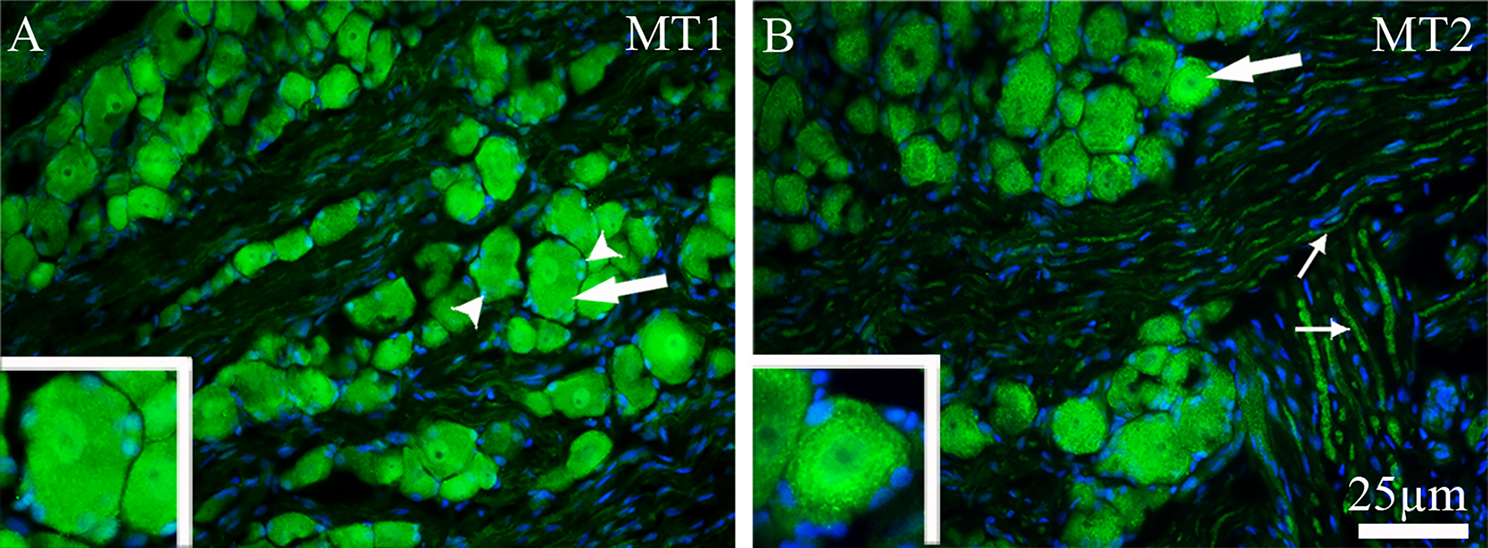
MT1 and MT2 immunoreactivity in the DRG. (**A**) MT1 expression in DRG was observed in both the cytoplasm and nuclei of neurons (thick arrow) and in SGCs (arrowheads). (**B**) MT2 immunoreactivity was localized in the cytoplasm of neurons and in some cases in their nuclei (thick arrow), and in the Aδ-fibers (thin arrows). The blue color represents nuclei staining with DAPI

Expression of MT1 and MT2 was also examined in another sensory ganglion involved in pain pathology, the DRG. As shown on Fig. 9, MT1 immunoreactivity was found in both the cytoplasm and nucleus of neurons, as well as in SGCs (Fig. 9A). In contrast, MT2 immunoreactivity was predominantly localized in the cytoplasm of neurons and in the Aδ-fibers (Fig. 9B).

### Expression and localization of melatonin receptors in cerebral arteries

**Fig. 10:**
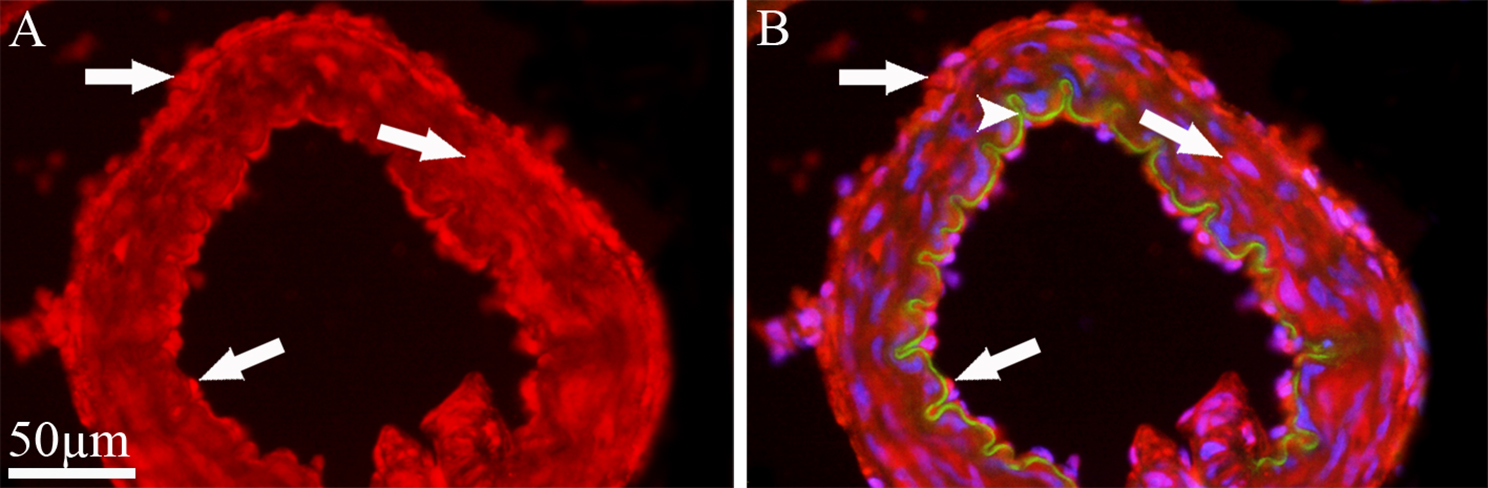
Immunohistochemistry of MT1 expression in the wall of cerebral arteries. (**A** and **B**) MT1 immunoreactivity (red) was observed in all layers of the basilar artery (thick arrows), i.e., in the adventitia layer and the endothelium as well as in the smooth muscle cells. Green represents the internal elastic lamina, autofluorescence (arrowhead in **B**) and blue represents nuclei staining with DAPI

Cerebral arteries are innervated by the trigeminal nerve and are a key component of the trigeminovascular system. Thus, potential localization of MT1 and MT2 receptors in male rat basilar artery was examined by immunohistochemistry. MT1 immunoreactivity was observed in all layers of cerebral vessels (Fig. 10).

This was mainly localized in the endothelium and in the adventitial layer as well as to some extent in the cytoplasm of smooth muscle cells (SMCs). In contrast, no expression of MT2 was detected in the walls of cerebral arteries.

## Discussion

The results of the current study clearly show that the sensory and parasympathetic ganglia, TG, SPG and DRG, are all direct targets of the pineal hormone melatonin. MT1 and MT2 receptors are present in neurons, and MT1 is additionally expressed in SGCs within the ganglia. The current findings indicate that peripheral sensory and parasympathetic processes can be regulated locally by the circadian system via melatonin signaling and suggest a mechanism by which circadian influences alter primary headache pathology.

### MT1 and MT2 receptors in the TG

Excitation of the trigeminal nerve is a key component driving the pain underlying severe headaches in migraine and cluster syndromes. Better understanding of the function and regulation of trigeminal neurons has led to development of effective treatments such as triptans, gepants and CGRP-related monoclonal antibodies [23]. Circadian aspects of trigeminal function are not well understood, but they are likely involved in the chronobiology of primary headache disorders [28]. TG, itself, expresses the molecular machinery to generate autonomous circadian rhythms. TG neurons contain core clock proteins such as Per2, BMAL1, and CRY2 [29–31]. Interestingly, clock genes also are expressed in SGCs [29]. Moreover, autonomous circadian rhythms of gene expression can be demonstrated in ex vivo cultures of the TG and its cells [29, 30]. These findings suggest local clock mechanisms can control timing of trigeminal activity but also indicate the need for synchronization with the master body clock in the hypothalamic SCN, which is a typical role for circulating melatonin [32, 33]. Our finding of MT1 and MT2 receptors within the TG supports this role. Since melatonin and related analogs are well known to shift and/or restore aberrant physiological rhythms [9, 32], TG melatonin receptors offer a target for regulating rhythms of TG activity.

Both melatonin receptor subtypes, MT1 and MT2, are expressed in the rat TG, as measured using RT-qPCR and protein immunohistochemistry. Several studies using rat pain models have also detected immunoreactivity for MT1 [34, 35] and MT2 [35] receptors in the TG. At the time of day studied in the current study, MT1 receptor mRNA had higher levels of expression relative to mRNA for MT2. No male-female differences in mRNA levels were seen for either MT1 or MT2 receptors. However, one caveat to keep in mind is that these findings represent a snapshot in time, i.e., the levels of gene expression and cellular localization that exist in normal rats at 8:00–9:00 o’clock in the morning, just prior to lights on and at a time when circulating levels of melatonin are low. We do not know if melatonin receptor expression in the TG varies over the circadian cycle.

### Receptor localization in the TG

MT1 and MT2 receptors showed distinct distribution patterns within the TG. MT1 immunoreactivity was observed in over half of all TG neurons; about 10% appeared to be C-fiber cell bodies containing CGRP [23, 36]. MT1 receptors also co-localized within a subset of neuronal cell bodies expressing RAMP1, an integral component of the CGRP receptor that is found in Aδ neurons [23, 36]. Thus, melatonin may affect CGRP signaling via MT1 receptors.

In contrast, MT2 melatonin receptors were primarily found in Aδ neuronal cell bodies and axons. Interestingly, TG expression of RAMP1, which is also found in Aδ cells, has been shown in mice to vary over the circadian cycle [29]. Together, these findings suggest MT2 receptors may regulate TG levels of CGRP receptors. MT2, but not MT1, receptors were detected not only in Aδ nerve fibers within the ganglion but also in the terminal regions of these nerves innervating the dura mater [37]. MT2 receptors mediate presynaptic inhibition of transmitter release in other pathways [24] and may have this effect on Aδ transmission. In addition, MT2 immunoreactivity was observed to co-express with CASPR in the paranodal regions at nodes of Ranvier along the Aδ-fibers. This intriguing finding suggests melatonin may influence Aδ-fiber excitability through nodal mechanisms, similar to what we previously described for CGRP receptors that are also located at the nodes [38].

MT2 receptors were found to be co-localized in only a small subset of small to medium-sized CGRP-positive neurons. Liu et al. did not observe MT2/CGRP co-localization in the TG of control rats, however, after induction of a temporomandibular joint osteoarthritis pain model, they found increased TG expression of CGRP that now included MT2-positive neurons [35]. TG levels of CGRP were also elevated in a nitroglycerin-induced migraine model, and this increase could be inhibited by melatonin acting via pharmacologically-identified MT2 receptors [39]. While more study is needed, these findings suggest an anti-nociceptive role for MT2 receptor to inhibit the increase in CGRP levels that occur during various types of pain, e.g., chronic, headache, inflammatory and neuropathic pain. Knockout of the MT2 receptor in mice resulted in lower thresholds for responding to nociceptive stimuli [40].

SGCs within TG also appear to contribute to effects of melatonin. These specialized cells are found in sensory ganglia in close association with neuronal cell bodies and are thought to be involved in inflammatory processing and sensitization of sensory nerves [23, 41]. We found MT1 melatonin receptor located on SGCs. Interestingly, these cells also express core clock genes which could drive local circadian rhythms of satellite glial activity [29, 31]. Circulating melatonin likely acts directly on these cells to align their rhythms with the master body clock in the SCN.

### MT1 and MT2 receptors in the SPG

In cluster headache, both the TG and the SPG are involved in the generation of intense headache pain and parasympathetic symptoms [6]. Due to the striking chronobiologic aspect of this disorder, we proposed that the SPG may also be regulated by melatonin. Indeed, both MT1 and MT2 melatonin receptors were found in neurons of the SPG. The pattern of neuronal expression was similar to that seen in the TG, that is, a more widespread cellular distribution of MT1 receptors and a restricted localization of MT2 receptors to medium-large neurons and axons. Co-localization studies indicate that melatonin can act on subsets of either VIP- or PACAP-containing neurons via either MT1 and/or MT2 receptors.

### MT1 and MT2 receptors in the DRG

We found a similar localization of MT1 and MT2 receptors in sensory neurons and SGCs in another rat sensory ganglion, the DRG. The TG and DRG have analogous roles in transmitting sensory information to the CNS, but from different parts of the body. Lin et al., also reported MT1 and MT2 immunoreactivity in mouse DRG [42], they also found MT1 receptors primarily located in SGCs while MT2 receptors were observed in neuronal cell bodies, often co-localized with CGRP. Reasons for differences in observed receptors distribution are not clear, but they could be related to the use of different antibodies and possible differences in time of day for tissue collection between the two studies.

### Melatonin receptors in cerebral arteries

A key target of the trigeminal nerve is the cerebral vasculature, particularly the large cerebral arteries on the surface of the brain. A long-standing theory of migraine proposed that headaches were initiated by vasodilation of the intracranial arteries, although recent views discount this idea [43]. Nevertheless, we examined the expression of melatonin receptors in rat basilar artery to provide a more comprehensive view of melatonin influences on the trigeminovascular system. MT1 receptor immunoreactivity was detected in all layers of the vessel wall, including the smooth muscle. The latter finding correlates well with previous studies of rat cerebral arteries that show MT1 mRNA expression [44] and functional contractile effects of melatonin [45, 46].

### Melatonin receptors in pain processing and primary headache

There is still much to learn about the specific effects of MT1 and MT2 activation in TG, SPG and DRG. Studies to date suggest melatonin inhibits nociceptive responses of these ganglia, in part by decreasing the electrical excitability of sensory neurons [35, 40, 42, 47, 48]. Both MT1 and MT2 receptors are G protein-coupled receptors that primarily associate with G_i/o_ proteins to cause a number of cellular effects, including inhibition of cAMP production and activation of inward rectifying potassium channels [24, 25]. The latter effect, in particular, may contribute to decreased neuronal excitability. In addition, melatonin agonists have been shown to suppress glial inflammation [49], which suggests MT1 receptors on satellite glia may mediate anti-inflammatory effects. It is likely that melatonin receptors also regulate local clock mechanisms within the ganglia, similar to their actions on the SCN master clock [33], however this remains to be demonstrated.

The identification of melatonin receptors within specific nerve and glial cells in sensory and parasympathetic ganglia supports the emerging view that melatonin is a factor in circadian/circannual variations in pain disorders. In addition to targeting these peripheral ganglia, melatonin acts on receptors in numerous brain regions involved in pain processing as well as on the master body clock in the hypothalamic SCN [32–34, 50, 51]. The underlying mechanisms and role of circadian dysfunction in primary headache are not known [6]. However, patients with migraine and cluster headache exhibit decreased nocturnal levels of circulating melatonin [2, 11, 12, 14]. Thus, nociceptive protection of the TG and SPG by this hormone would be diminished at this time and could lower the threshold for headache onset. Moreover, disruption of the melatonin rhythm would impact the ability of this hormone to synchronize local rhythms in sensory nerves and satellite glia in the TG and SPG and align them with the master clock in the hypothalamus [33]. Interestingly, both the TG and SPG directly innervate the pineal gland [52, 53] and thus pathologic changes in the ganglia could affect melatonin rhythms. The pineal gland contains CGRP receptors [54] and, as it is located outside the blood-brain barrier, it is another potential target for therapeutic CGRP-related gepants and antibodies. A number of clinical studies suggest melatonin agonists are a promising novel approach to treatment of primary headache disorders [10, 14–20, 55]. More study is needed to determine which melatonin receptor to target as well as the proper time of administration [9] to optimize circadian and circannual regulation of headache onset.

## Limitations

A limitation of the present study is the lack of knockout validation for the MT1 and MT2 antibodies. While multiple antibodies from different suppliers produced consistent and reproducible staining patterns, none have been tested in MTNR1A or MTNR1B knockout tissue, which represents the most definitive method for confirming specificity. Additionally, our study does not include physiological or circadian expression data, which could provide further insight into the temporal regulation of MT1 and MT2 in trigeminal and sphenopalatine ganglia. Future studies incorporating genetic knockout models and time-of-day analyses will be important to address these aspects and further validate our findings.

## Conclusions

In conclusion, this study demonstrates that MT1 and MT2 receptors are expressed in the TVS and SPG, key components involved in migraine and cluster headache. MT1 is more widely expressed than MT2, notably in neurons, SGCs, and cerebral vessels, suggesting a prominent role in modulating trigeminal and vascular functions. Melatonin receptor co-localization with neuropeptides involved in autonomic and sensory neuronal regulation supports a potential mechanism by which melatonin influences headache onset and progression. Together, these findings support a role for melatonin influence in primary headache pathophysiology and point to its potential for novel headache treatment.

## Electronic supplementary material

Below is the link to the electronic supplementary material.

Supplementary material 1

## Data Availability

The datasets generated during and/or analyzed during the current study are available from the corresponding author on reasonable request.

## Abbreviations

BA: Basilar artery
BSA: Bovine serum albumin
CASPR: Contactin-associated protein 1
CGRP: Calcitonin gene-related peptide
CNS: Central nervous system
cDNA: Complementary DNA
DRG: Dorsal root ganglion
DAPI: 4’, 6-diamidino-2-phenylindole
DNA: Deoxyribonucleic acid
GAPDH: Glyceraldehyde-3-phosphate dehydrogenase
mRNA: Messenger RNA
MT1: Melatonin receptor 1
MT2: Melatonin receptor 2
PBS: Phosphate buffer saline
PBS-T: Phosphate buffer saline-TritonX
PF: Paraformaldehyde
PACA: Pituitary adenylate cyclase activating polypeptide
RNA: Ribonucleic acid
RT-qPCR: Real-time quantitative polymerase chain reaction
SGC: Satellite glial cell
SPG: Sphenopalatine ganglion
SMC: Smooth muscle cell
TVS: Trigeminovascular system
TG: Trigeminal ganglion
VIP: Vasoactive intestinal peptide
