# Supplementary figures and images for: Expression of melatonin receptors in trigeminal and sphenopalatine ganglia: potential targets for primary headache disorders
Source: J Headache Pain. 2025 Dec 9;26(1):284. doi: 10.1186/s10194-025-02215-9 (PMC12690866; doi:10.1186/s10194-025-02215-9)

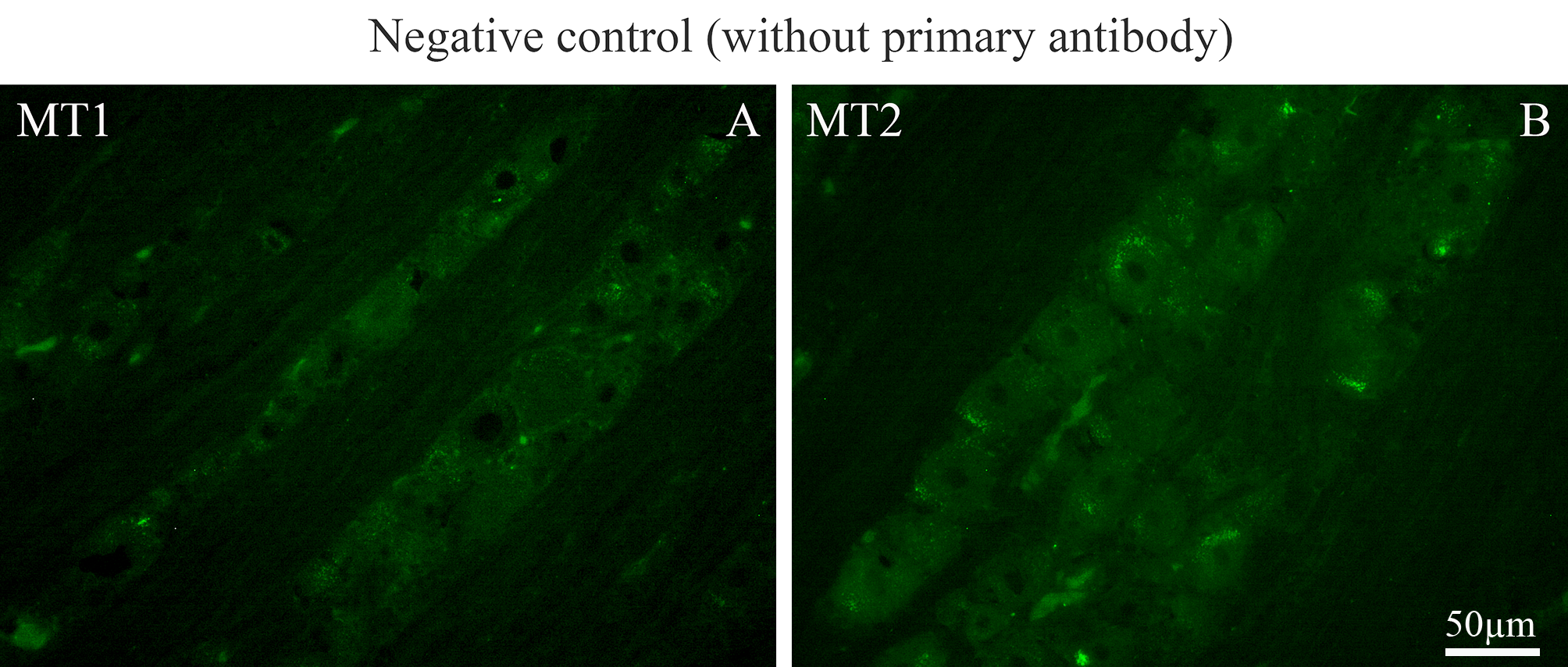

Supplement: Supplementary file 1 — Supplementary material 1 [file 10194_2025_2215_MOESM1_ESM.tif]
